# Supplementary material for: S-1-propenyl-L-cysteine suppresses lipopolysaccharide-induced expression of matrix metalloproteinase-1 through inhibition of tumor necrosis factor-α converting enzyme-epidermal growth factor receptor axis in human gingival fibroblasts
Source: PLoS One. 2023 Apr 21;18(4):e0284713. doi: 10.1371/journal.pone.0284713 (PMC10121056; doi:10.1371/journal.pone.0284713)
Supplement: S1 Raw images — The contrast and brightness of these blots are modulated in the manuscript. (PDF) [file pone.0284713.s002.pdf]

Supplementary Information
– S1 Raw images –

PLOS ONE

***S*-1-propenyl-L-cysteine suppresses lipopolysaccharide-induced expression of matrix metalloproteinase-1 through inhibition of tumor necrosis factor-α converting enzyme-epidermal growth factor receptor axis in human gingival fibroblasts**

Hiroshi Nango and Masahiro Ohtani

Central Research Institute, Wakunaga Pharmaceutical Co., Ltd., 1624 Shimokotachi, Koda-Cho, Akitakata, Hiroshima 739-1195, Japan

Corresponding author
nangou_h@wakunaga.co.jp (HN)

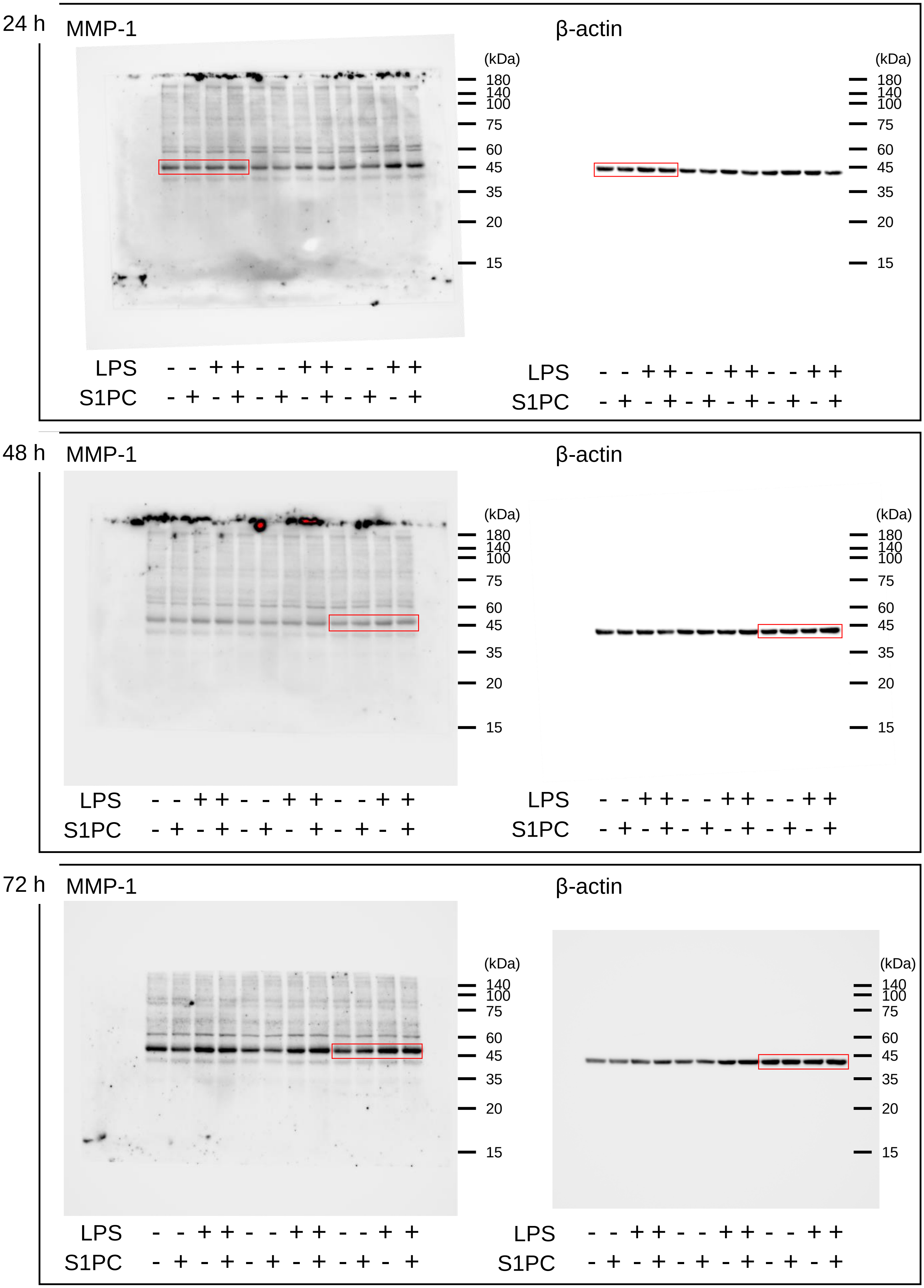

Full-width of the membranes of the original blots used in Fig. 2c. The contrast and brightness of these blots are modulated in the manuscript.

a

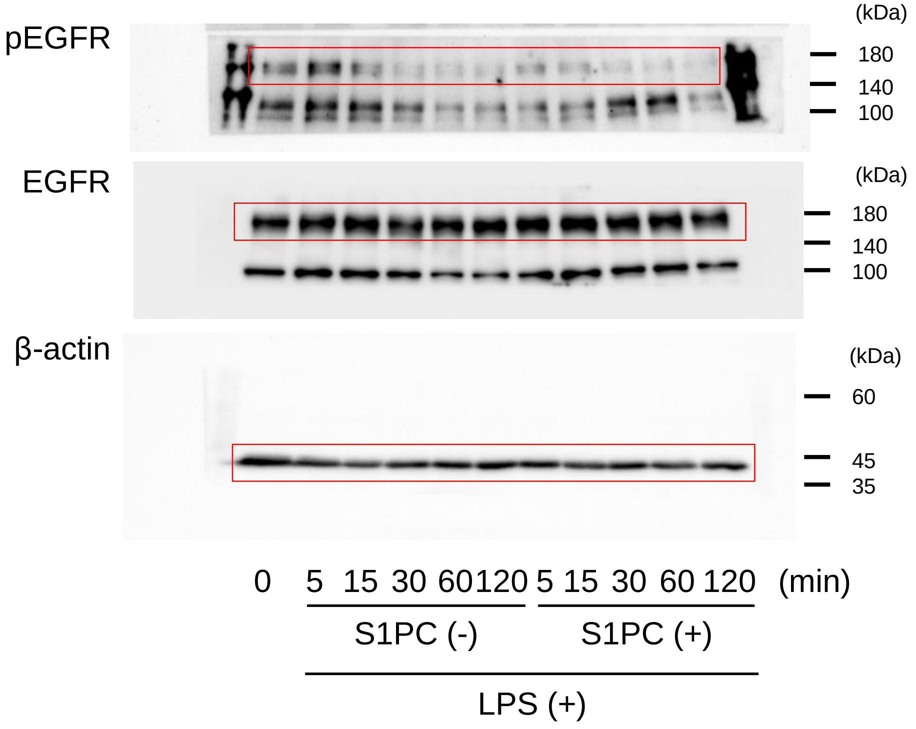

b

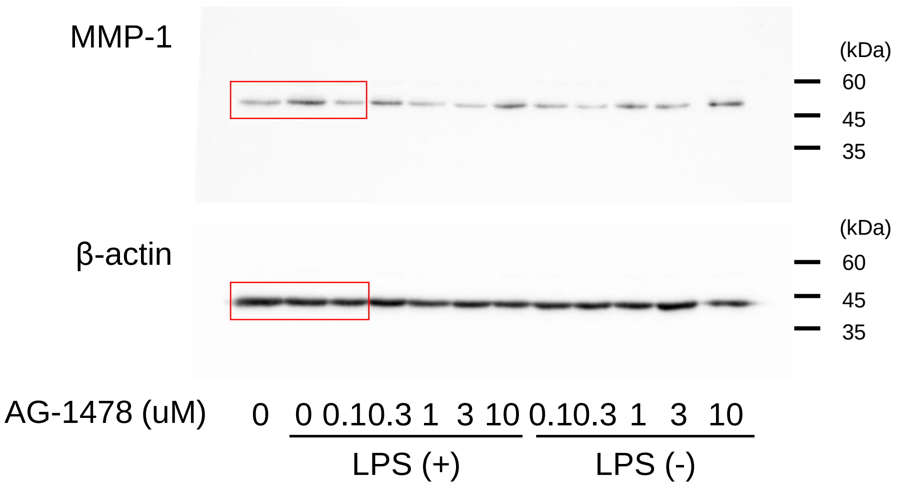

Full-width of the membranes of the original blots used in Fig. 3a (**a**) and 3b (**b**). The contrast and brightness of these blots are modulated in the manuscript.

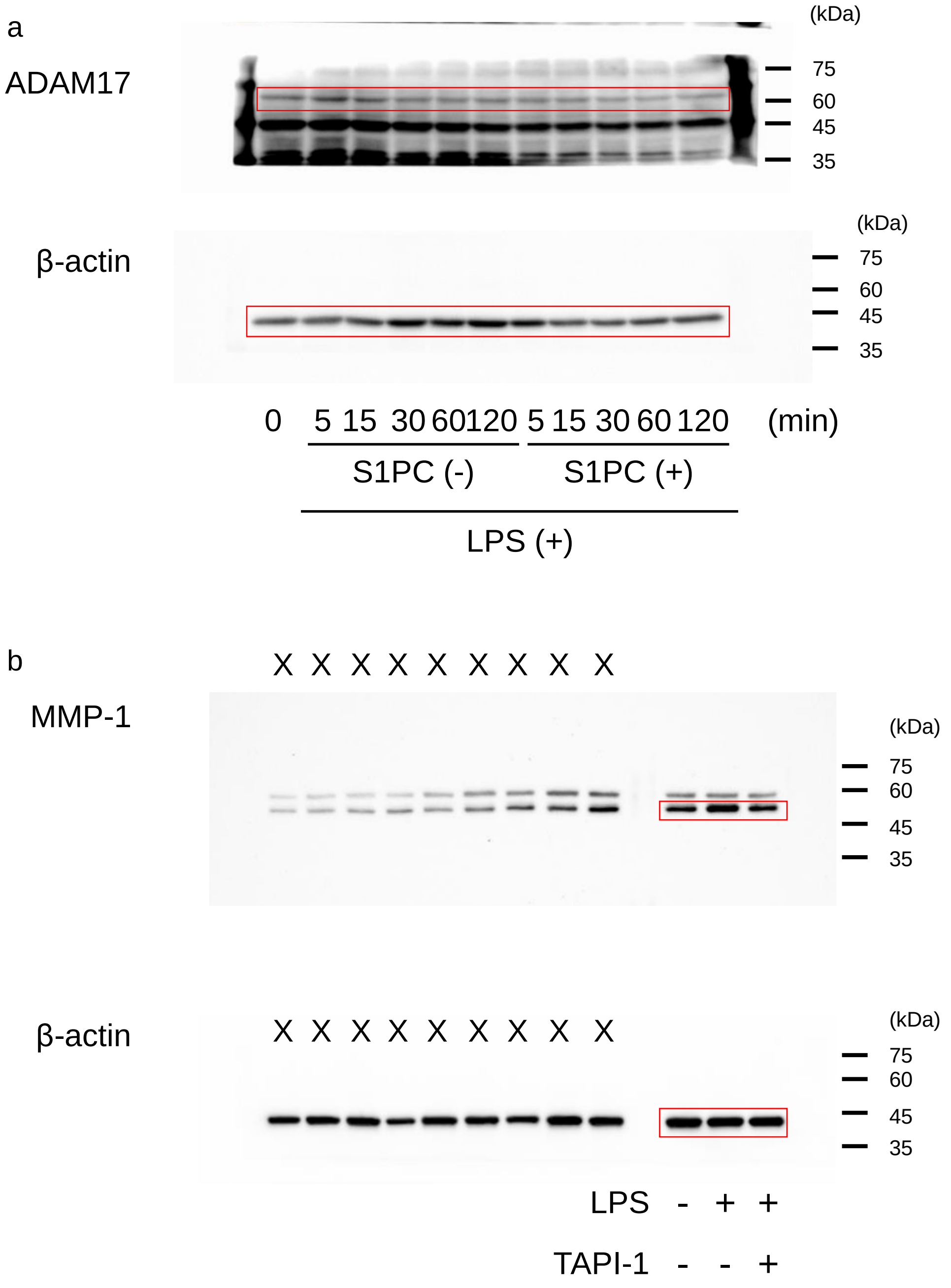

Full-width of the membranes of the original blots used in Fig. 4a (**a**) and 4b (**b**). The contrast and brightness of these blots are modulated in the manuscript.
